# A single model for glioblastoma segmentation with and without T2-FLAIR: independent validation of a targeted dropout strategy

**DOI:** 10.3389/fneur.2026.1889198

**Published:** 2026-07-28

**Authors:** Marco Öchsner, Lena Kaiser, Robert Stahl, Nathalie L. Albert, Thomas Liebig, Robert Forbrig, Jonas Reis

**Affiliations:** 1 Institute of Neuroradiology, LMU University Hospital, LMU, Munich, Germany; 2 Department of Nuclear Medicine, LMU University Hospital, LMU, Munich, Germany; 3 German Cancer Consortium (DKTK), Partner Site Munich, Munich, Germany; 4 Bavarian Cancer Research Center (BZKF), Munich, Germany

**Keywords:** fluid-attenuated inversion recovery, independent validation, missing imaging sequences, tumor segmentation, volumetry

## Abstract

**Objectives:**

To evaluate targeted T2 fluid-attenuated inversion recovery (T2-FLAIR) dropout for robust automated glioblastoma segmentation and volumetry when T2-FLAIR is unavailable, while preserving performance when the full MRI protocol is available.

**Materials and methods:**

In this retrospective multi-dataset study, 3D nnU-Net models were developed on BraTS 2021 after excluding UPenn-GBM cases (remaining *n* = 848) and evaluated in the independent withheld UPenn-GBM cohort (*n* = 403). Models were trained with or without targeted T2-FLAIR dropout by zeroing the T2-FLAIR channel during training. Testing used prespecified T2-FLAIR-present and T2-FLAIR-unavailable scenarios, where the unavailable scenario was simulated by zeroing the T2-FLAIR channel at inference. The primary endpoint was per-patient overall region-wise Dice similarity coefficient (DSC). Secondary endpoints were region-specific DSC, 95th percentile Hausdorff distance and Bland–Altman whole-tumor volume bias.

**Results:**

In the UPenn-GBM validation cohort, performance was preserved with the full MRI protocol: overall median DSC was 94.8% [interquartile range (IQR) 90.0–97.1%] with 35% dropout and 95.0% (IQR 90.3–97.1%) without dropout. In the T2-FLAIR-unavailable scenario, targeted dropout improved overall median DSC from 81.0% (IQR 75.1–86.4%) to 93.4% (IQR 89.1–96.2%). Whole-tumor DSC improved from 60.4 to 92.6%, whole-tumor 95th percentile Hausdorff distance from 17.24 mm to 2.45 mm, and whole-tumor volume bias from −45.6 mL to 0.83 mL. A dedicated three-sequence nnU-Net achieved similar performance without T2-FLAIR (overall DSC 93.8%, WT DSC 93.5%), suggesting that much of this recovery reflects adaptation to the reduced-input setting rather than dropout training specifically.

**Conclusion:**

In the independent withheld UPenn-GBM cohort, targeted T2-FLAIR dropout preserved complete-protocol performance and remained robust when T2-FLAIR was unavailable. Because a dedicated three-sequence model matched this performance without T2-FLAIR, the value of targeted dropout lies not in superior missing-sequence accuracy but in providing a single model that operates across both complete and T2-FLAIR-unavailable inference.

## Introduction

Glioblastoma is the most common primary malignant brain tumor in adults and requires MRI assessment of contrast-enhancing and non-enhancing components for treatment planning and response assessment (1, 2). Because manual delineation of tumor compartments is time-consuming and subject to inter-reader variability, reliable automated tumor segmentation is clinically attractive (3–5). Deep learning methods, particularly U-Net-style architectures and self-configuring models such as nnU-Net, have achieved strong performance and are increasingly used as the standard tool for brain tumor segmentation (6, 7). The Multimodal Brain Tumor Segmentation (BraTS) challenges have accelerated this development through large, multi-institutional multiparametric MRI datasets with standardized annotations (8, 9). However, most high-performing pipelines still require a complete four-sequence structural MRI protocol [T1-weighted, post-contrast T1-weighted (T1-CE), T2-weighted, and T2-fluid attenuated inversion recovery (T2-FLAIR)] (4, 6) despite the fact that retrospective and real-world clinical data are often incomplete, heterogeneous, or partially unusable.

This deployment gap matters because the likelihood of missing data increases with the number of required inputs, and prior work suggests that whole-tumor segmentation depends strongly on T2/FLAIR information (10–12). Absent T2-FLAIR therefore merits study in its own right, rather than as one instance of a generic missing-modality problem. Although RANO 2.0 assigns less weight to non-enhancing disease in many *IDH*-wildtype glioblastoma response-assessment settings, T2/FLAIR remains relevant for characterizing non-enhancing abnormality and edema and for imaging contexts in which non-enhancing disease is clinically consequential, including antiangiogenic treatment (2, 13). From a segmentation perspective, unavailable or non-diagnostic T2-FLAIR may therefore distort whole-tumor volumetry even when the other structural sequences are available. Several strategies have been proposed to address missing MRI modalities, including synthesis of unavailable sequences using generative models (11, 14, 15), heteromodal representation learning (16), and sequence dropout training (17, 18). Among these, targeted sequence dropout is appealing for clinical deployment because it does not require an additional synthesis model or separate models for each possible input subset. The intended use of the present approach is robust segmentation-driven tumor delineation and whole-tumor volumetry when T2-FLAIR is missing or unusable in retrospective or heterogeneous clinical MRI, rather than replacement of diagnostic image interpretation or a universal solution to all missing-modality scenarios. What remains insufficiently defined is whether targeted T2-FLAIR dropout can preserve segmentation performance under the full four-sequence protocol while preventing clinically meaningful failure when T2-FLAIR is unavailable in an independent withheld test cohort. We therefore developed models on BraTS 2021 after excluding UPenn-GBM and evaluated all primary study endpoints in the withheld UPenn-GBM cohort.

The primary contribution of this study is not a new segmentation architecture. Rather, we evaluate a targeted T2-FLAIR dropout strategy within a standard 3D nnU-Net framework and quantify its effect on segmentation accuracy, boundary error, and whole-tumor volumetric agreement under complete and T2-FLAIR-unavailable inference conditions. To distinguish targeted dropout from simple adaptation to a reduced-input setting, we trained a dedicated three-sequence nnU-Net using T1, T1-CE, and T2 only. An additional TumorSynth comparison serves as contextual benchmarking against a broader robustness-oriented segmentation strategy.

## Materials and methods

### Study design and reporting

This retrospective multi-dataset study analyzed only publicly available, de-identified MRI datasets. Accordingly, ethics approval and informed consent were not required, and the study involved no interaction with human participants. Reporting was aligned with the Metrics Reloaded recommendations for image analysis validation, the Checklist for Artificial Intelligence in Medical Imaging (CLAIM) and its 2024 update (19, 20), with a completed checklist provided in Supplementary material.

### Cohort and reference standard

**Figure 1:**
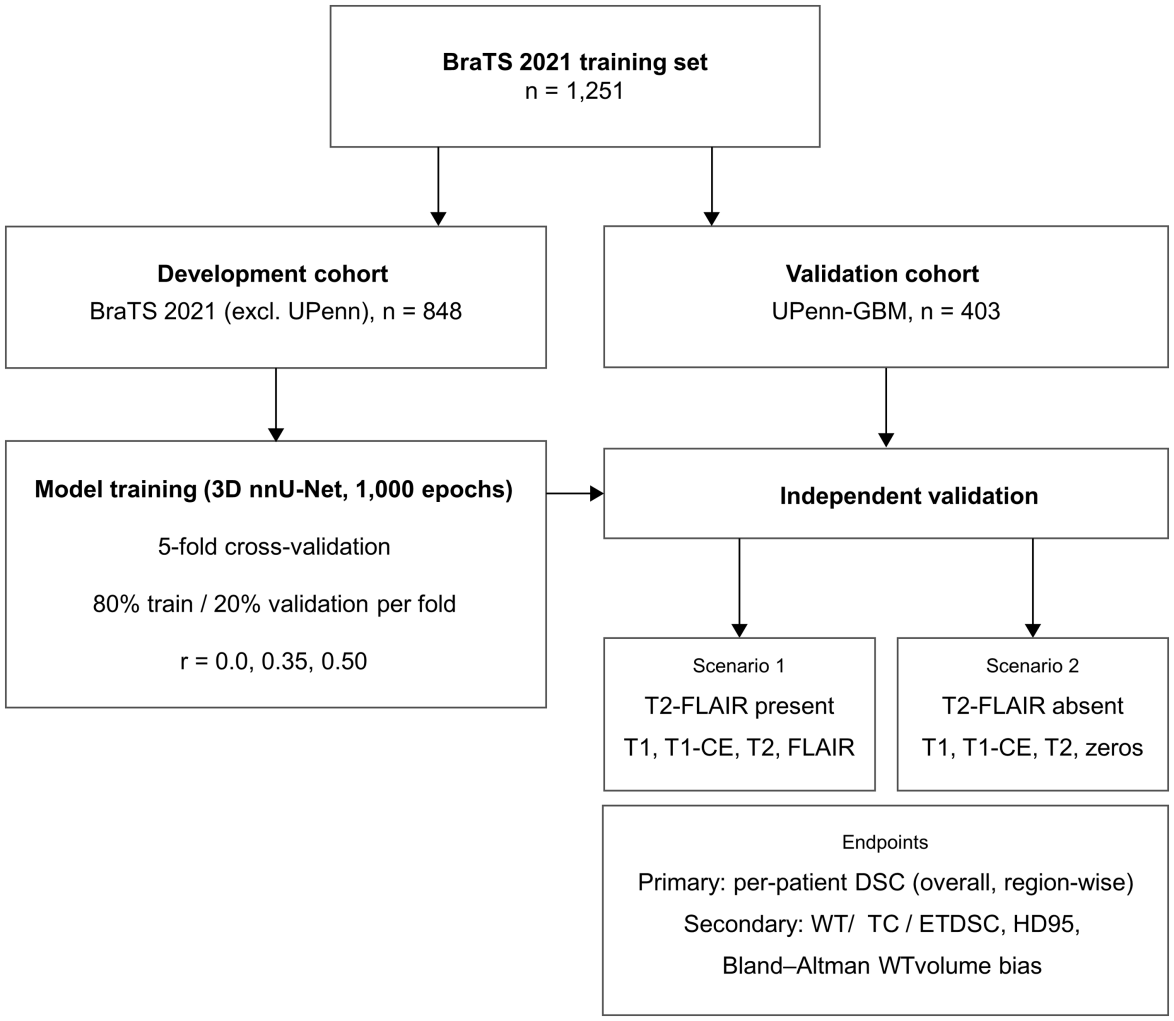
Study design and evaluation workflow. BraTS 2021 cases excluding UPenn were used for five-fold cross-validation training of 3D nnU-Net models with targeted T2-FLAIR dropout rates of 0.0, 0.35, and 0.50; final testing used the held-out UPenn-GBM cohort under two prespecified inference scenarios: T2-FLAIR present and T2-FLAIR unavailable (T2-FLAIR channel replaced with zeros). The primary endpoint was per-patient overall region-wise Dice similarity coefficient (DSC); secondary endpoints were region-wise whole-tumor (WT), tumor core (TC), and enhancing tumor (ET) DSC, 95th percentile Hausdorff distance (HD95), and Bland–Altman WT volume bias. DSC, Dice similarity coefficient; ET, enhancing tumor; FLAIR, fluid-attenuated inversion recovery; HD95, 95th percentile Hausdorff distance; TC, tumor core; WT, whole-tumor; *r*, dropout rate.

Model development used the RSNA-ASNR-MICCAI BraTS 2021 training cohort after excluding the University of Pennsylvania glioblastoma cohort (UPenn-GBM, remaining BraTS *n* = 848) (9, 21). The released BraTS 2021 images had been skull stripped, co-registered, resampled to 1-mm isotropic resolution, and annotated by the dataset creators. The independent test cohort was UPenn-GBM (*n* = 403), which was withheld entirely from model development and used only for final evaluation (21, 22); the workflow is illustrated in Figure 1. We included all cases with (i) an available structural MRI including at least T1, T1-CE, T2, and T2-FLAIR, and (ii) an available reference standard segmentation. In both cohorts, the reference standard segmentation comprised necrotic/non-enhancing tumor (NCR/NET), peritumoral edema (ED), and enhancing tumor (ET) provided by the dataset creators; no study-specific manual contour editing was performed (9, 21). Because UPenn-GBM is part of the BraTS/UPenn public-data ecosystem and uses standardized preprocessing, we refer to this as independent UPenn-GBM testing rather than validation on unrelated routine clinical DICOM data.

### Segmentation targets and inference scenarios

Two output configurations were evaluated. Region-wise outputs were prespecified for the main analyses because they correspond more directly to clinically interpretable tumor compartments: whole-tumor (WT = NCR/NET ∪ ED ∪ ET), tumor core (TC = NCR/NET ∪ ET), and enhancing tumor (ET). Label-wise outputs following the original BraTS convention (NCR/NET, ED, ET) were analyzed as supportive secondary outputs. Each test case was evaluated under two prespecified inference scenarios: T2-FLAIR-present (T1, T1-CE, T2, and T2-FLAIR) and T2-FLAIR-unavailable. The unavailable-sequence condition was simulated by replacing the preprocessed T2-FLAIR channel with zeros immediately before inference. This paired design isolates the effect of unavailable T2-FLAIR while holding anatomy, reference segmentation, and the remaining input sequences constant. It was chosen to evaluate the deployment setting defined above. No synthetic image generation was used, and no attempt was made to model degraded or artifact-contaminated T2-FLAIR.

### Model development

All models were developed with the 3D full-resolution nnU-Net framework (7). Six model configurations were trained: {label-wise, region-wise} × {T2-FLAIR dropout rate (r) = 0.0, 0.35, 0.50}. For the T2-FLAIR dropout models, only the T2-FLAIR channel was replaced with zeros during training, with probability r per training sample, while T1, T1-CE, and T2 were never dropped. Training used patient-level 5-fold cross-validation (80% training/20% validation within each fold) on the BraTS 2021 development cohort with nnU-Net default 3D full-resolution settings and 1,000 epochs per fold. For each fold, the checkpoint with the highest mean foreground Dice similarity coefficient on the validation partition was selected, and cross-validation ensemble learning was used for inference. Fold-level validation was used solely for model optimization and ensemble construction and was not treated as a separate study endpoint. The no-dropout setting, *r* = 0.0, represented standard complete-protocol training. The *r* = 0.35 setting was selected *a priori* as the primary targeted-dropout configuration because it repeatedly exposed the model to T2-FLAIR-unavailable samples while retaining complete-protocol training in most iterations. The *r* = 0.50 setting was included as a sensitivity analysis representing equal exposure to complete and T2-FLAIR-unavailable inputs. No UPenn-GBM case was used to select dropout rates, train models, tune hyperparameters, or choose checkpoints.

To distinguish the effect of targeted dropout from adaptation to a reduced-input distribution, a dedicated three-sequence nnU-Net was trained using only T1, T1-CE, and T2, with the same development cohort, five-fold cross-validation design, nnU-Net configuration, training schedule, checkpoint-selection rule, and independent UPenn-GBM test cohort as the primary models. This model has a single inference mode and does not receive a T2-FLAIR channel. A contrast- and resolution-agnostic comparator (TumorSynth) was additionally evaluated as contextual benchmarking (23). Paired differences between each comparator and the targeted-dropout model were summarized with 95% percentile-bootstrap confidence intervals (CI); no confirmatory superiority or equivalence hypothesis was assigned to these comparator analyses.

### Statistical analysis

Analyses were organized hierarchically to control multiplicity by design rather than by *post hoc* correction. One confirmatory family was prespecified: equivalence of targeted dropout (*r* = 0.35) versus no dropout (*r* = 0.0) in the T2-FLAIR-present scenario, tested by two one-sided tests (TOST) across the four region-wise endpoints (overall, WT, TC, ET) (24). Within this family, the Holm procedure was applied to control the family-wise error rate at *α* = 0.05; because all equivalence *p*-values were <0.001, conclusions were identical with and without correction. The primary robustness comparison, targeted dropout versus no dropout in the T2-FLAIR-unavailable scenario, was a single prespecified estimation summarized by effect size with a 95% CI. All remaining analyses, region-specific (WT, TC, ET) and label-wise DSCs (NCR/NET, ED, ET) DSCs, 95th percentile Hausdorff distance (HD95), Bland–Altman volume bias, the *r* = 0.50 configuration, the dedicated three-sequence nnU-Net model, and the TumorSynth comparison, were prespecified as secondary or exploratory; interpretation rests on effect sizes with 95% CIs, and any accompanying *p*-values are descriptive, uncorrected for multiplicity, and not used for confirmatory inference. DSC and HD95 were summarized as medians with interquartile ranges because patient-level segmentation metrics are bounded and commonly skewed. For paired model comparisons, effect sizes are reported as median paired differences with 95% CIs from a nonparametric percentile bootstrap using 2,000 patient-level resamples with replacement, with paired observations resampled at the patient level. Whole-tumor volumetric agreement was evaluated with Bland–Altman analysis as predicted minus reference whole-tumor volume in milliliters, and in accordance with Bland–Altman convention, systematic bias was reported as the arithmetic mean paired difference with 95% bootstrap confidence intervals and limits of agreement.

The equivalence margin was prespecified at ±1.5 DSC percentage points. No consensus equivalence margin exists for DSC in brain tumor segmentation, and we therefore selected a deliberately small margin and contextualized it against the known reproducibility limits of manual glioma segmentation. In prior work, expert manual glioma segmentations showed imperfect spatial overlap even under structured conditions, with reported generalized conformity index values (a Jaccard-family overlap index monotonically related to DSC) of 0.79 preoperatively and 0.32 postoperatively for glioblastoma enhancing-tumor segmentation, and 0.77 and 0.48 for preoperative and postoperative expert non-glioblastoma segmentation, respectively (3). Although these values are not DSC margins directly, they indicate that human reference annotations carry non-negligible spatial variability. A ± 1.5 DSC percentage-point margin is therefore small relative to the reported scale of inter-rater variation and was considered unlikely to alter qualitative clinical interpretation when boundary error and volumetric agreement remained stable. The margin is also substantially smaller than the performance shift expected in the T2-FLAIR-unavailable scenario, providing a conservative threshold for declaring complete-protocol performance preserved. All analyses were performed in Python 3.12 using PyTorch 2.8.0, NumPy 2.1.3, SciPy 1.15.3, and pandas 2.2.3.

## Results

**Figure 2:**
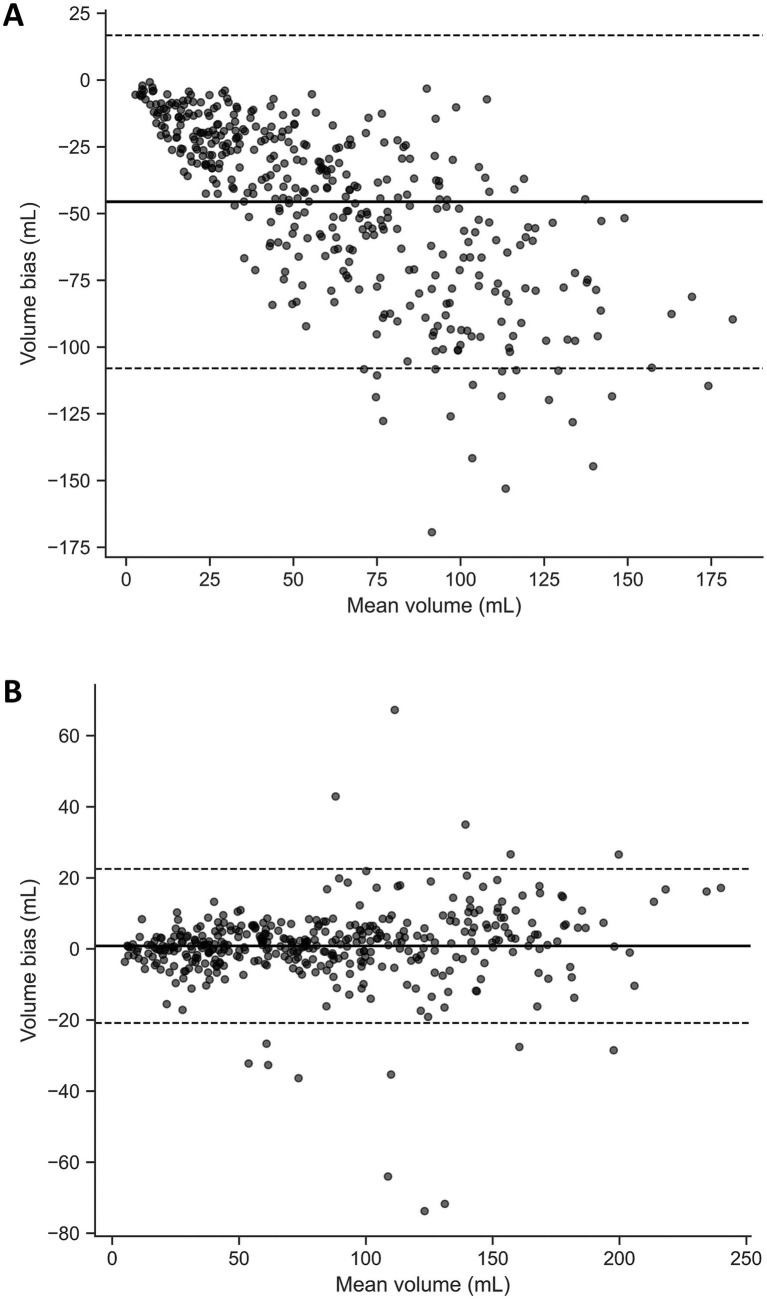
UPenn-GBM validation cohort (*n* = 403): Restoration of whole-tumor volumetric agreement with T2-FLAIR unavailable. Bland–Altman plots of predicted minus reference standard whole-tumor volume (mL) in the T2-FLAIR-unavailable scenario for the region-wise nnU-Net trained **(A)** without dropout (*r* = 0.0; Bias: −45.59 mL; Limits of Agreement: −107.96 to 16.78 mL) and **(B)** with targeted T2-FLAIR dropout (*r* = 0.35; Bias: 0.83 mL, Limits of Agreement: −20.83 to 22.49 mL). Solid lines indicate mean bias; dashed lines indicate 95% limits of agreement. FLAIR, fluid-attenuated inversion recovery; *r*, dropout rate.

**Figure 3:**
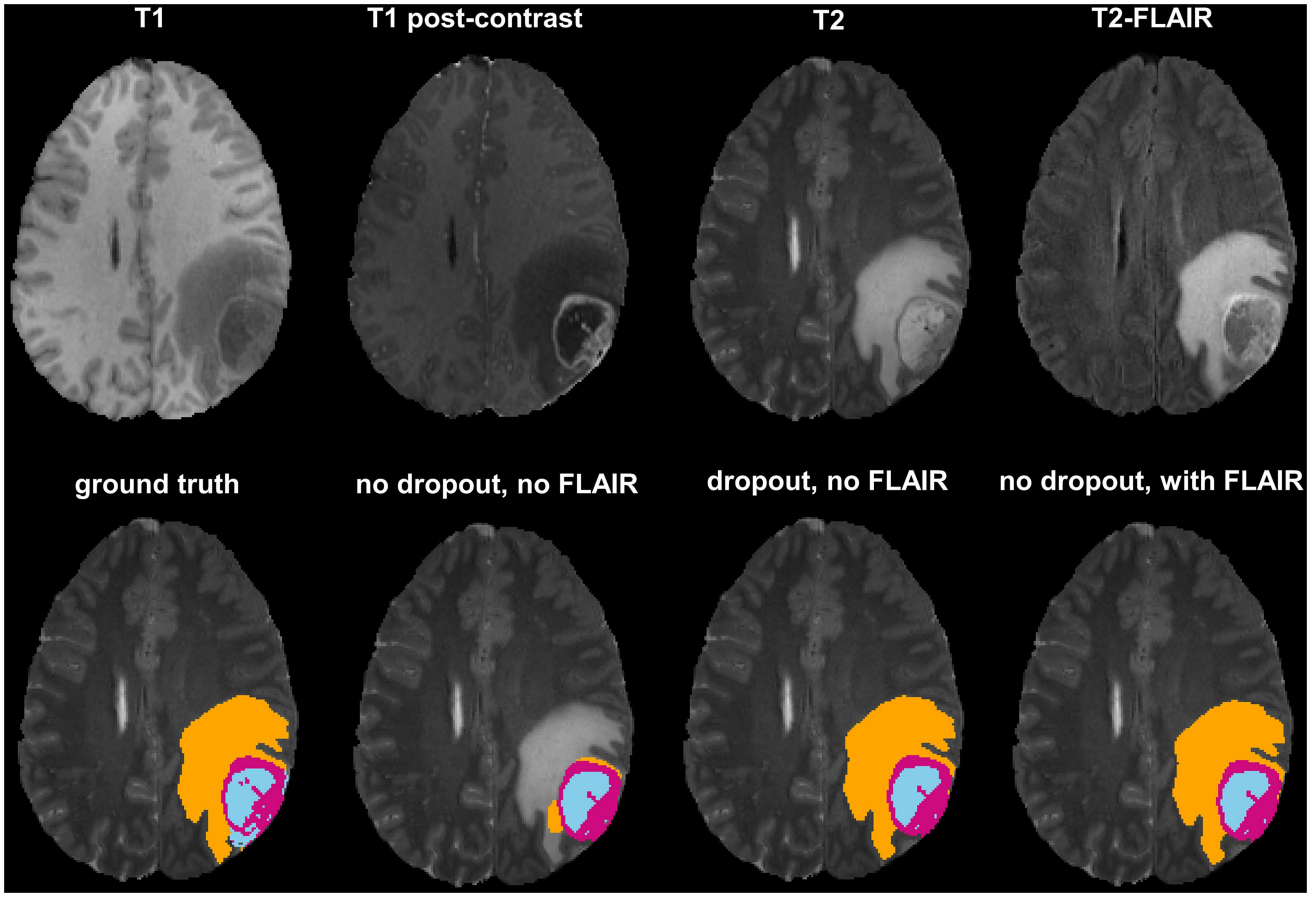
Representative UPenn-GBM case. Top row shows T1-weighted, post-contrast T1-weighted, T2-weighted, and T2-FLAIR images. Bottom row shows the reference standard, the region-wise nnU-Net prediction without dropout (*r* = 0.0) in the T2-FLAIR-unavailable scenario, the prediction with targeted T2-FLAIR dropout (*r* = 0.35) in the same scenario, and the no-dropout prediction in the T2-FLAIR-present scenario. This case illustrates whole-tumor undersegmentation without dropout when T2-FLAIR is unavailable and recovery with targeted dropout, corresponding to the cohort-level volumetric effect summarized in Figure 2. FLAIR, fluid-attenuated inversion recovery; *r*, dropout rate.

**Figure 4:**
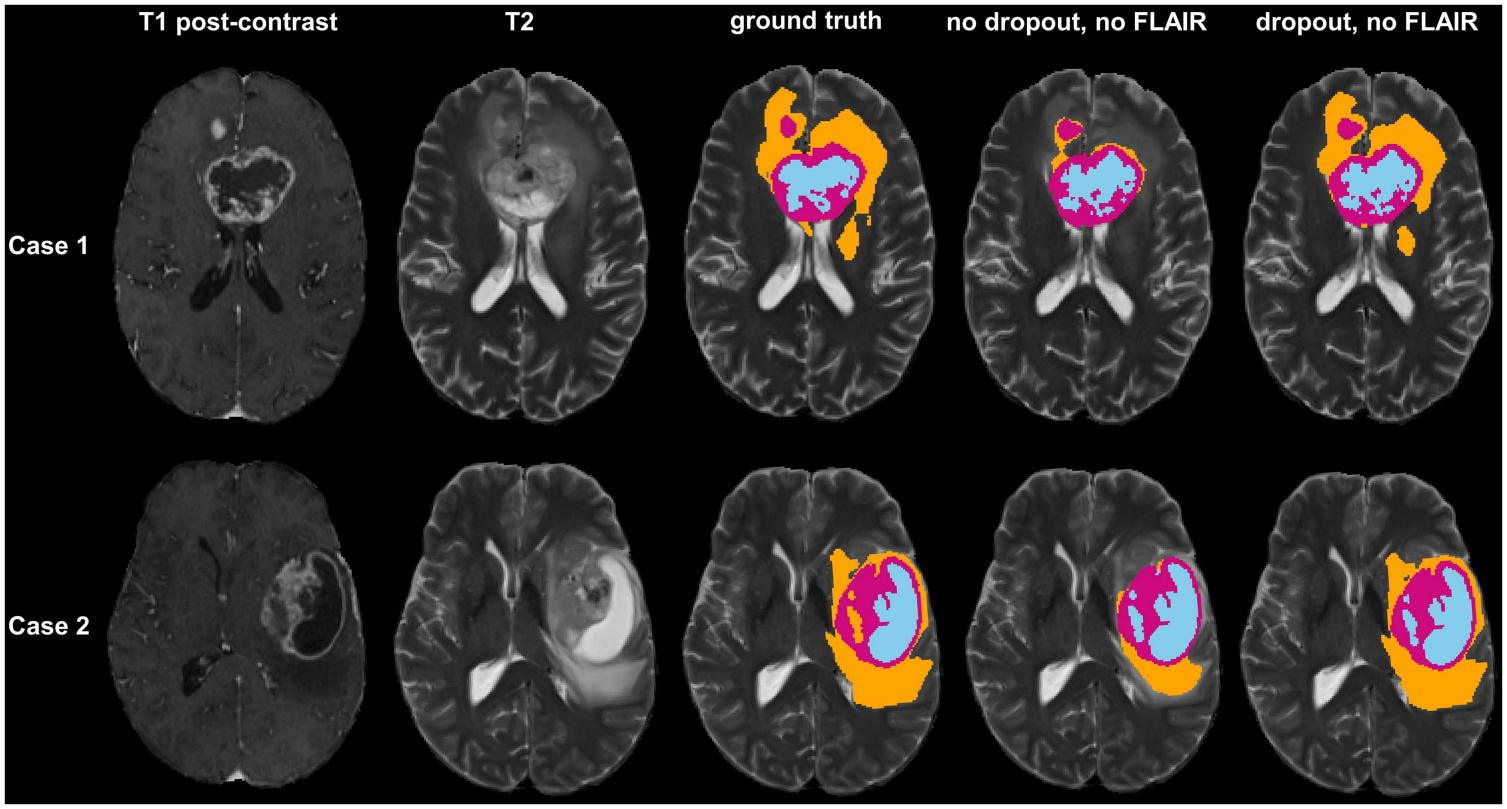
Representative UPenn-GBM cases. For each case, post-contrast T1-weighted and T2-FLAIR images are shown together with the reference standard, the region-wise nnU-Net prediction without dropout (*r* = 0.0) in the T2-FLAIR-unavailable scenario, and the prediction with targeted T2-FLAIR dropout (*r* = 0.35) in the same scenario. In the T2-FLAIR-unavailable columns, the T2-FLAIR channel was replaced with zeros at inference. These cases illustrate recurrent edema and whole-tumor undersegmentation without dropout and recovery with targeted dropout, corresponding to the cohort-level whole-tumor volumetric bias shown in Figure 2. FLAIR, fluid-attenuated inversion recovery; *r*, dropout rate.

Results are reported as median [Q1–Q3] across patients. All primary analyses were performed in the withheld UPenn-GBM test cohort. Region-wise model results are summarized in Tables 1, 2 and Figures 2–4. Figure 2 quantifies whole-tumor volumetric agreement, and Figures 3, 4 show representative UPenn-GBM cases. The comparative model analyses (three-sequence nnU-Net and TumorSynth) are provided in Tables 3, 4.

**Table 1:** UPenn-GBM validation cohort (*n* = 403): region-wise segmentation performance with T2-FLAIR present and unavailable at inference.

T2-FLAIR drop for training	Overall		WT		ET		TC	
	DSC med [Q1–Q3]	HD95 med [Q1–Q3]	DSC med [Q1–Q3]	HD95 med [Q1–Q3]	DSC med [Q1–Q3]	HD95 med [Q1–Q3]	DSC med [Q1–Q3]	HD95 med [Q1–Q3]
Segmentation with T2-FLAIR present								
*r* = 0.0	95.0 [90.3–97.1]	1.61 [1.14–2.72]	95.5 [91.9–97.8]	1.73 [1.00–3.32]	94.1 [87.6–96.8]	1.00 [1.00–1.41]	96.5 [92.9–98.1]	1.41 [1.00–2.45]
*r* = 0.35	94.8 [90.0–97.1]	1.55 [1.14–2.74]	95.6 [91.9–97.8]	1.62 [1.00–3.32]	94.0 [87.5–96.8]	1.00 [1.00–1.41]	96.4 [92.9–98.0]	1.41 [1.00–2.45]
*r* = 0.5	94.8 [90.2–97.0]	1.61 [1.14–2.71]	95.3 [91.9–97.7]	1.73 [1.00–3.46]	93.7 [87.4–96.7]	1.00 [1.00–1.41]	96.5 [92.8–98.1]	1.41 [1.00–2.45]
Segmentation with T2-FLAIR unavailable								
*r* = 0.0	81.0 [75.1–86.4]	7.59 [5.31–11.21]	60.4 [45.7–71.9]	17.24 [11.66–24.20]	92.4 [86.3–95.6]	1.00 [1.00–1.73]	94.4 [89.8–96.7]	2.45 [1.73–4.58]
*r* = 0.35	93.4 [89.1–96.2]	2.02 [1.41–3.41]	92.6 [88.7–95.2]	2.45 [1.73–5.00]	93.5 [87.5–96.4]	1.00 [1.00–1.41]	96.2 [92.4–97.8]	1.73 [1.00–2.83]
*r* = 0.5	93.7 [89.1–96.2]	2.03 [1.41–3.46]	93.0 [89.4–95.7]	2.24 [1.41–4.64]	93.7 [87.4–96.5]	1.00 [1.00–1.41]	96.2 [92.5–97.8]	1.73 [1.00–2.83]

DSC, Dice similarity coefficient (reported as %); HD95, 95th percentile Hausdorff distance (mm); WT, whole-tumor; TC, tumor core; ET, enhancing tumor; FLAIR, fluid-attenuated inversion recovery; r = dropout rate. Performance of region-wise nnU-Net models (WT, TC, ET) trained on BraTS 2021 with targeted T2-FLAIR dropout rates of 0.0, 0.35, and 0.50 and evaluated under two prespecified inference scenarios: T2-FLAIR present (all four sequences provided) and T2-FLAIR unavailable (T2-FLAIR channel replaced with zeros). Values are median [Q1–Q3] across patients. Overall denotes the unweighted macro-average across WT, TC, and ET.

**Table 2:** UPenn-GBM validation cohort (*n* = 403): paired *r* = 0.35 versus *r* = 0.0 in region-wise models.

Segmentation labels	Median DSC r(0.35)-r(0.0) [95%CI]	TOST *p*-value
Segmentation with T2-FLAIR present (confirmatory equivalence)		
Overall	−0.017 [−0.046, 0.014]	<0.001
ET	−0.04 [−0.094, −0.006]	<0.001
TC	−0.011 [−0.040, 0.015]	<0.001
WT	−0.005 [−0.029, 0.023]	<0.001
Segmentation with T2-FLAIR unavailable (descriptive robustness effect)		
Overall	11.35 [10.63, 12.21]	**–**
ET	0.75 [0.663, 0.837]	**–**
TC	1.12 [0.97, 1.29]	**–**
WT	31.1 [28.2, 33.48]	**–**

TOST, two one-sided tests; DSC, Dice similarity coefficient; WT, whole-tumor; TC, tumor core; ET, enhancing tumor; FLAIR, fluid-attenuated inversion recovery; r, dropout rate. Entries report median paired DSC differences (*r* = 0.35 – *r* = 0.0) in percentage points with 95% percentile-bootstrap confidence intervals. In the T2-FLAIR-present scenario, complete-protocol equivalence was assessed by two one-sided tests within prespecified bounds of ±1.5 DSC percentage points (*α* = 0.05), Holm-corrected across the overall, WT, TC, and ET endpoints (confirmatory family). In the T2-FLAIR-unavailable scenario, targeted dropout was evaluated for robustness improvement, not equivalence; differences are therefore reported descriptively without hypothesis testing. Differences are expressed in DSC percentage points throughout: The near-zero present-scenario values reflect preserved complete-protocol performance, and the large positive absent-scenario values reflect the robustness gain from targeted dropout.

**Table 3:** UPenn-GBM validation cohort (*n* = 403): comparator segmentation performance in the T2-FLAIR-unavailable scenario.

Model (no T2-FLAIR)	Overall DSC	WT DSC	ET DSC	TC DSC	WT HD95	WT bias (mL)
Targeted dropout (*r* = 0.35)	93.4 [89.1–96.2]	92.6 [88.7–95.2]	93.5 [87.5–96.4]	96.2 [92.4–97.8]	2.45 [1.73–5.00]	0.83
Dedicated three-sequence	93.8 [89.3–96.3]	93.5 [89.9–95.7]	93.6 [87.4–96.6]	96.3 [92.9–97.9]	2.24 [1.41–4.25]	0.77
TumorSynth	79.6 [66.1–85.7]	88.4 [83.4–91.3]	69.3 [47.0–78.4]	85.5 [75.1–90.5]	5.00 [3.61–8.09]	3.19

DSC, Dice similarity coefficient (%); HD95, 95th percentile Hausdorff distance (mm); WT, whole tumor; TC, tumor core; ET, enhancing tumor; FLAIR, fluid-attenuated inversion recovery; r = dropout rate. Region-wise targeted-dropout (*r* = 0.35), a dedicated three-sequence nnU-Net (T1, T1-CE, T2), and TumorSynth, all evaluated without T2-FLAIR. The three-sequence model has a single inference mode and no T2-FLAIR channel; TumorSynth values are the no-FLAIR condition. DSC and HD95 are median [Q1–Q3] across patients; whole-tumor volume bias is the mean paired difference (predicted minus reference). Overall denotes the unweighted macro-average across WT, TC, and ET.

**Table 4:** UPenn-GBM validation cohort (*n* = 403): complete-protocol cost of T2-FLAIR-blindness.

Compartment	Median ΔDSC (pp)	95% CI
Edema	−2.87	−3.26, −2.65
Whole tumor	−1.74	−1.87, −1.61
Tumor core	−0.24	−0.30, −0.20
Enhancing tumor	−0.02	−0.11, 0.03

DSC, Dice similarity coefficient; pp, percentage points; CI, confidence interval; FLAIR, fluid-attenuated inversion recovery. Median paired DSC differences (dedicated three-sequence model minus targeted-dropout model evaluated with T2-FLAIR present), in percentage points with 95% percentile-bootstrap confidence intervals. The three-sequence model has no T2-FLAIR channel and therefore cannot use T2-FLAIR when available. Negative values indicate lower three-sequence performance. Differences are descriptive; no equivalence or superiority hypothesis was tested.

### Performance with T2-FLAIR present

In the independent test cohort, when T2-FLAIR was available, targeted T2-FLAIR dropout training preserved segmentation performance relative to standard four-sequence training (Table 1). For the region-wise models, overall DSC was 95.0% (90.3–97.1) without dropout and 94.8% (90.0–97.1) with targeted dropout (*r* = 0.35), with comparable boundary error [HD95 1.61 mm (1.14–2.72) vs. 1.55 mm (1.14–2.74)]. Region-specific performance was similarly stable, including WT DSC of 95.5% versus 95.6%, ET DSC of 94.1% versus 94.0%, and TC DSC of 96.5% versus 96.4% for *r* = 0.0 and *r* = 0.35, respectively. Equivalence of *r* = 0.35 versus *r* = 0.0 within ±1.5 DSC percentage points was supported across endpoints in the T2-FLAIR-present setting (Holm-corrected TOST *p* < 0.001 throughout) (Table 2). The realized differences were below 0.05 percentage points across all region-wise endpoints, well inside the prespecified margin.

### Performance and whole-tumor volumetric agreement in the simulated T2-FLAIR-unavailable scenario

In the same test cohort, in the simulated T2-FLAIR-unavailable scenario, performance deteriorated substantially without robustness training, whereas targeted dropout largely prevented this failure (Table 1). Overall DSC fell to 81.0% (75.1–86.4) without dropout, and the deterioration was concentrated in whole-tumor segmentation: WT DSC fell to 60.4% (45.7–71.9) and WT HD95 rose to 17.24 mm (11.66–24.20) without dropout, whereas targeted dropout (*r* = 0.35) maintained WT DSC at 92.6% (88.7–95.2) and WT HD95 at 2.45 mm (1.73–5.00). Bland–Altman analysis showed marked systematic whole-tumor underestimation without dropout, with mean volume bias (predicted minus reference standard) of −45.6 mL [95% CI, −48.8– (−42.6) mL] and wide limits of agreement from −108.0 to 16.8 mL (Figure 2A). The *r* = 0.35 dropout model largely eliminated this bias, with a mean volume bias of 0.83 mL (95% CI, −0.28–1.92 mL) and tighter limits of agreement from −20.8 to 22.5 mL (Figure 2B). Representative qualitative examples of this failure mode and its recovery are shown in Figures 3, 4. By contrast, ET and TC segmentation were comparatively stable, with ET DSC of 92.4% versus 93.5% and TC DSC of 94.4% versus 96.2% without versus with targeted dropout. Supportive label-wise analyses localized this failure mode to edema, with marked recovery under targeted dropout (Supplementary Table S1). Overall, targeted dropout improved median overall DSC by 11.4 percentage points (95% CI, 10.6–12.2) relative to no-dropout training in this scenario; this comparison was prespecified to quantify robustness improvement rather than equivalence, and effect sizes are reported descriptively with bootstrap confidence intervals (Table 2).

### Comparison with a dedicated three-sequence nnU-net and with TumorSynth

To determine whether the FLAIR-unavailable robustness reflected targeted dropout specifically or adaptation to a reduced-input setting, the *r* = 0.35 targeted-dropout model was compared with a dedicated three-sequence nnU-Net trained only on T1, T1-CE, and T2. In the T2-FLAIR-unavailable scenario, the three-sequence model performed similarly to targeted dropout, with overall DSC of 93.8% (89.3–96.3), WT DSC of 93.5% (89.9–95.7), ET DSC of 93.6% (87.4–96.6), and TC DSC of 96.3% (92.9–97.9); WT HD95 was 2.24 mm (1.41–4.25) and mean WT volume bias was 0.77 mL (limits of agreement, −23.3 to 24.8 mL) (Table 3). Paired differences between the two models in this scenario were small (median WT difference 0.59 percentage points; overall 0.25 percentage points). The two strategies diverged under complete-protocol inference. Because the three-sequence model has no T2-FLAIR channel, it cannot use T2-FLAIR when it is available. Compared with the targeted-dropout model evaluated with T2-FLAIR present, the three-sequence model showed lower DSC concentrated in the FLAIR-associated compartments: median paired differences were −2.87 percentage points for edema and −1.74 for whole tumor, versus −0.02 for enhancing tumor and −0.24 for tumor core (Table 4).

TumorSynth, a contrast- and resolution-agnostic comparator, showed lower DSC than the nnU-Net-based models in both conditions. Overall DSC was 81.9% (71.4–86.9) with T2-FLAIR and 79.6% (66.1–85.7) without. WT DSC was 90.2% (85.4–92.6) and 88.4% (83.4–91.3), respectively. Enhancing-tumor and tumor-core DSC were lower than for the targeted-dropout and three-sequence models (Table 3). WT volume bias was 0.04 mL (limits of agreement, −25.5 to 25.6 mL) with T2-FLAIR and 3.19 mL (−25.1 to 31.5 mL) without, with wider limits of agreement than the nnU-Net-based models throughout. These values are reported as contextual benchmarking; because TumorSynth was designed for contrast- and resolution-agnostic segmentation rather than this specific missing-sequence task.

### Supplementary analyses

Results for the alternative dropout configuration (*r* = 0.50), supportive label-wise analyses, additional paired equivalence testing, and contextual benchmarking against HD-GLIO are provided in Supplementary Tables S1–S5 and were consistent with the main findings.

## Discussion

In this retrospective multi-dataset study with independent UPenn-GBM testing, targeted T2-FLAIR dropout preserved glioblastoma segmentation performance under the complete four-sequence MRI protocol and largely prevented the pronounced FLAIR-unavailable failure mode observed with standard four-sequence training. The dedicated three-sequence comparator refines the interpretation of this finding. A model trained only on T1, T1-CE, and T2 performed similarly to targeted dropout in the T2-FLAIR-unavailable scenario, indicating that adaptation to the reduced-input setting, rather than dropout training specifically, accounts for most of this recovery. The dedicated three-sequence model is therefore a strong FLAIR-unavailable specialist, but one that forgoes recoverable T2-FLAIR information on complete-protocol cases, with the largest loss in the FLAIR-associated edema and whole-tumor compartments. The practical value of targeted dropout therefore lies in its deployment profile: it approaches FLAIR-unavailable specialist performance while retaining a single model that also uses T2-FLAIR when available.

These findings align with and extend prior work on missing-modality brain tumor segmentation. Existing strategies include modality synthesis, heteromodal representation learning, and sequence dropout training (11, 14, 16, 25, 26). Targeted dropout is narrower than synthesis-based or broadly heteromodal approaches, but this narrower scope is also its practical advantage: it addresses a prespecified missing-sequence scenario within a standard nnU-Net pipeline without requiring a separate synthesis network or multiple subset-specific deployment models (18). The dedicated three-sequence model shows that a modality-specific reduced-input model can perform very well when T2-FLAIR is unavailable, but such a model necessarily discards T2-FLAIR in complete-protocol cases and requires a separate deployment pathway. Recent work in pediatric brain tumors and contrast−/resolution-agnostic segmentation frameworks such as TumorSynth further underscores that incomplete and heterogeneous MRI input is a substantive deployment problem rather than a technical edge case (23, 27). The TumorSynth comparison further supports focused robustness training for this specific FLAIR-unavailable setting, as TumorSynth showed lower absolute segmentation accuracy despite its broader contrast- and resolution-agnostic design. This comparison should be interpreted as contextual benchmarking, not as a direct head-to-head comparison. TumorSynth and the nnU-Net-based targeted-dropout model were designed with different objectives, preprocessing assumptions, and intended deployment scenarios. TumorSynth targets contrast- and resolution-agnostic segmentation across arbitrary inputs, whereas our model targets a single prespecified missing-sequence pattern within a standardized pipeline. Differences in absolute accuracy therefore reflect divergent design goals as much as performance, and should not be read as evidence of general superiority. TumorSynth also illustrates a measurement caveat relevant to volumetric endpoints: although its whole-tumor volume bias was small in absolute terms, its wider limits of agreement and lower overlap metrics indicate that near-zero mean volume bias need not reflect spatially accurate segmentation, because opposing voxel-level errors can cancel in a volumetric summary, reinforcing the value of reporting DSC, HD95, and Bland–Altman agreement jointly. Conversely, no single metric adequately reflects clinical usability: in the present study the concordant improvement across DSC, HD95, and Bland–Altman volume bias under targeted dropout supports the interpretation that the observed gain was clinically meaningful rather than merely numerical.

This pattern is neuroradiologically plausible. T2-weighted imaging carries substantial information about edema and non-enhancing abnormality, and a model trained explicitly without T2-FLAIR can learn to recover surrogate whole-tumor cues from T2, T1, and post-contrast T1-weighted imaging. A standard four-sequence model trained only on complete inputs may instead become dependent on the expected T2-FLAIR channel and fail when that channel is replaced by zeros. Targeted modality dropout addresses this by repeatedly exposing the network to T2-FLAIR-unavailable inputs during training, encouraging reliance on redundant information in the remaining contrasts while preserving access to T2-FLAIR when it is present. The relative stability of enhancing-tumor segmentation across conditions is consistent with its stronger dependence on post-contrast T1-weighted imaging (2, 9, 10, 13).

The value of a single robustness-trained model is most apparent when protocol completeness cannot be assumed or verified. An alternative deployment strategy would select between a complete-protocol model and a dedicated three-sequence model according to T2-FLAIR availability. Such conditional model selection can match single-model accuracy when sequence availability is known exactly, but it depends on reliable identification of usable T2-FLAIR and retains the catastrophic failure mode of the complete-protocol model whenever a present-but-non-diagnostic T2-FLAIR is mistakenly treated as usable. A single model trained with targeted dropout avoids this dependence: it requires no parallel model maintenance or input-conditional routing, and it degrades gracefully rather than catastrophically when T2-FLAIR is absent, incomplete, or of uncertain quality. Its practical advantage is therefore operational robustness in heterogeneous workflows rather than higher peak accuracy than a modality-specific specialist. These considerations suggest a simple deployment heuristic. Where T2-FLAIR is consistently available, a standard four-sequence model remains the natural choice; where it is consistently absent, a dedicated three-sequence model is an appropriate and comparably accurate option. The single targeted-dropout model offers its greatest advantage in the intermediate and most common case: heterogeneous workflows in which T2-FLAIR is present for some cases but missing or non-diagnostic in others and protocol completeness cannot be verified in advance. There it avoids both the catastrophic failure of a four-sequence model when T2-FLAIR is unavailable and the discarding of T2-FLAIR by a three-sequence model when it is present.

Preserving access to T2-FLAIR when it is available is clinically consequential, because T2-FLAIR abnormality remains relevant for pretreatment characterization, delineation of edema and non-enhancing abnormality, radiotherapy planning, and whole-tumor volumetry (13). This broader relevance is also consistent with recent amino-acid PET literature: PET RANO 1.0 formalized PET-based response assessment in diffuse gliomas, and in newly diagnosed glioblastoma, [^18^F]FET PET frequently identifies metabolically active tumor burden that extends beyond MRI contrast enhancement and may remain measurable when MRI-RANO does not show measurable disease (28, 29). The most immediate application of the present approach is therefore robust segmentation-driven volumetry in retrospective datasets, multicenter studies, and heterogeneous clinical workflows, rather than replacement of diagnostic image interpretation.

This study has several limitations. First, the T2-FLAIR-unavailable condition was simulated by zeroing an otherwise available preprocessed channel. This paired design isolates complete modality loss while holding anatomy, reference segmentation, and the remaining sequences constant, but it does not capture degraded-yet-present T2-FLAIR acquisitions, including motion corruption, incomplete coverage, registration error, or abnormal intensity distributions. In clinical deployment, a degraded T2-FLAIR image might still influence the model unless sequence-level quality control identifies it as non-diagnostic and routes inference to a FLAIR- unavailable mode. The present results are therefore most directly applicable to absent or intentionally excluded T2-FLAIR, not to all forms of degraded-FLAIR imaging. Second, although UPenn-GBM was independent and withheld from model development, it remained a single-institution, multi-scanner cohort drawn from the broader BraTS/UPenn public dataset. In addition, both the development and test cohorts were distributed after standardized BraTS-style preprocessing, including skull stripping, co-registration, and resampling to 1-mm isotropic resolution, with similar acquisition protocols. Hence, generalizability to other acquisition parameters will need to be further examined. Third, the analysis was restricted to a targeted missingness pattern and to baseline pretreatment glioblastoma; robustness to other missing sequences, combinations of missing and degraded inputs, postoperative examinations, and follow-up imaging remains unproven. Fourth, the reference standard was derived from public expert annotations, and no study-specific contour adjudication or inter-reader variability analysis was performed. Fifth, the dedicated three-sequence nnU-Net performed similarly to targeted dropout in the T2-FLAIR-unavailable scenario. The present results therefore do not establish targeted dropout as a superior FLAIR-unavailable specialist; rather, they support it as a single-model strategy that preserves complete-protocol operation while approaching the comparable accuracy of a modality-specific reduced-input model. Where T2-FLAIR is consistently unavailable, a dedicated three-sequence model may be an appropriate alternative; in mixed retrospective datasets, multicenter studies, and workflows with variable or uncertain protocol completeness, a single targeted-dropout model may reduce the need for parallel model maintenance and input-conditional routing.

Consequently, future work should evaluate the approach under other missingness and degradation patterns, across unrelated routine clinical cohorts from additional institutions and acquisition protocols, including cases with genuinely missing or non-diagnostic T2-FLAIR. Such comparisons would also help clarify the practical trade-off between a single robustness-trained model and modality-specific deployment strategies.

In the independent withheld UPenn-GBM cohort, targeted T2-FLAIR dropout preserved complete-protocol segmentation performance and largely prevented the FLAIR-unavailable failure mode of standard four-sequence training. A dedicated three-sequence nnU-Net achieved similar performance without T2-FLAIR, indicating that targeted dropout is not a superior FLAIR-unavailable specialist; its value instead lies in providing a single model that operates across complete and T2-FLAIR-unavailable inference. These findings support targeted sequence dropout as a practical robustness strategy for automated glioblastoma analysis in heterogeneous clinical workflows in which T2-FLAIR may be missing or unusable.

## Data Availability

The original contributions presented in the study are included in the article/Supplementary material, further inquiries can be directed to the corresponding author.
