# Supplementary material for: A single model for glioblastoma segmentation with and without T2-FLAIR: independent validation of a targeted dropout strategy
Source: Front Neurol. 2026 Jul 28;17:1889198. doi: 10.3389/fneur.2026.1889198 (PMC13457126; doi:10.3389/fneur.2026.1889198)
Supplement: Supplementary file 1 [file Table_1.docx]

**Supplementary File: CLAIM Checklist (2024 Update)**

Manuscript: **Robust Glioblastoma Segmentation and Volumetry Without T2-FLAIR: External Validation of Targeted Dropout Training**

| **CLAIM item** | **Checklist requirement (paraphrased)** | **Addressed** | **Manuscript location** | **Notes** |
| --- | --- | --- | --- | --- |
| 1 | Identify the work as an AI/ML study in the title/abstract. | Yes | Title; Abstract header and text |  |
| 2 | Provide a structured abstract that summarizes design, data, model, evaluation, and main results. | Yes | Abstract (Purpose/Materials and Methods/Results/Conclusion) |  |
| 3 | Describe clinical/scientific background and intended use/role of the AI tool. | Yes | Introduction |  |
| 4 | State study objective(s) and/or hypothesis(es). | Yes | End of Introduction (Purpose and hypotheses) |  |
| 5 | Specify whether the study is prospective or retrospective. | Yes | Methods – Study design and reporting |  |
| 6 | State study goal (e.g., model development, validation, noninferiority/equivalence, etc.). | Yes | Introduction, Methods |  |
| 7 | Describe data sources (origin, dataset name(s), public/private, recruitment). | Yes | Methods – Datasets and reference standard (BraTS 2021; UPenn‑GBM) |  |
| 8 | Define inclusion/exclusion criteria and study setting/time frame. | Yes | Methods – Datasets and reference standard |  |
| 9 | Describe preprocessing steps applied to the data. | Yes | Methods – Datasets and reference standard | BraTS organizer preprocessing is described |
| 10 | Describe any selection of subsets/regions/patches/frames for model input. | Yes | Methods – Training and testing | Extraction of UPenn GBM dataset for external validation dataset is described |
| 11 | Describe de-identification and privacy protection approach. | Yes | Methods – Study design and reporting | Only public de‑identified datasets are used |
| 12 | Describe missing data and how it was handled. | NA | Methods | Full public dataset was used, which only contains subjects with full four modality imaging set |
| 13 | Provide imaging acquisition protocol details (scanner, sequences, key parameters) or cite where available. | Partial | Methods | BraTS is a heterogenous imaging dataset, parameters for each site are publicly available and referenced |
| 14 | Identify and justify the reference standard used. | Yes | Methods |  |
| 15 | Describe how reference annotations/labels were generated. | Yes | Methods |  |
| 16 | Define what constitutes a positive case / target definition(s). | Yes | Methods | Tumor subcompartments and derived regions are defined |
| 17 | Describe reference standard quality assurance (expertise, blinding, adjudication). | Yes | Methods |  |
| 18 | Report inter- and/or intra-rater variability (and/or mitigation) for the reference standard. | NA |  | Ground truth segmentations obtained from BraTS dataset |
| 19 | Describe data partitioning for training/validation/testing (and sizes). | Yes | Methods |  |
| 20 | State the level of disjointness between partitions (e.g., patient-level). | NA |  |  |
| 21 | Describe how sample size was determined (power/precision or rationale). | Partial | Methods | No formal sample size calculation was performed, as all available data were used |
| 22 | Describe model architecture; cite prior architecture if used; describe modifications. | Yes | Methods | nnU‑Net 3D full-resolution used, with region- and label-based training, with and without FLAIR-dropout at fixed proportions |
| 23 | Report software/frameworks and versions; provide access information when applicable. | Yes | Methods |  |
| 24 | Describe model parameter initialization (including randomness/seed control). |  |  |  |
| 25 | Describe training procedure and hyperparameters (optimizer, LR, epochs, augmentation, loss, early stopping). | Partial | Methods | Epochs and use of nnU‑Net defaults are stated; consider adding a concise hyperparameter summary or nnU‑Net plan/config file in Supplementary Methods. |
| 26 | Describe how the final model/checkpoint was selected. | Yes | Methods |  |
| 27 | Describe any ensembling and how outputs were combined. | Yes | Methods |  |
| 28 | Specify evaluation metrics and rationale/clinical relevance. | Yes | Methods – Statistics |  |
| 29 | Describe statistical analysis and uncertainty estimation. | Yes | Methods – Statistics |  |
| 30 | Describe robustness/sensitivity analyses. | Yes | Methods - Statistics |  |
| 31 | Describe model explainability/interpretability methods (if used). | NA |  |  |
| 32 | Report performance on internal data (training/validation/test as applicable). | NA |  |  |
| 33 | Report performance on external data (if applicable). | Yes | Results; Supplementary |  |
| 34 | Provide clinical trial registration information (if applicable). | NA |  | Retrospective analysis of public de‑identified datasets; no registration expected. |
| 35 | Provide participant/data flow (numbers included/excluded) per cohort. | Yes | Methods |  |
| 36 | Report participant demographics/clinical characteristics (when available). | Partial | Methods | BraTS demographics are briefly described and cited |
| 37 | Report performance metrics with measures of uncertainty (CI, IQR) and appropriate aggregation. | Yes | Results and Supplementary | Medians/IQRs and bootstrap CIs for paired differences are provided. |
| 38 | Report subgroup analyses (if performed) and pre-specification. | NA |  |  |
| 39 | Provide analysis of errors/failures (qualitative or quantitative). | Yes | Results/Discussion |  |
| 40 | Report interpretability outputs/results (if interpretability methods used). | NA |  |  |
| 41 | Discuss limitations, biases, and generalizability. | Yes | Discussion – Limitations paragraph |  |
| 42 | Discuss clinical implications and future work. | Yes | Discussion; Conclusion |  |
| 43 | State availability of data, code, and/or trained model (or restrictions). | No | Data availability statement |  |
| 44 | Report funding sources and role of funders. | Yes | Funding statement | . |
