# Supplementary material for: A single model for glioblastoma segmentation with and without T2-FLAIR: independent validation of a targeted dropout strategy
Source: Front Neurol. 2026 Jul 28;17:1889198. doi: 10.3389/fneur.2026.1889198 (PMC13457126; doi:10.3389/fneur.2026.1889198)
Supplement: Supplementary file 2 [file Table_2.docx]

Supplementary Material

**Supplementary Tables**

|  | **Overall** | | **Enhancing Tumor** | | **Non-enhancing Tumor** | | **Edema** | |
| --- | --- | --- | --- | --- | --- | --- | --- | --- |
| T2-FLAIR drop for training | DSC med [Q1-Q3] | HD95 med [Q1-Q3] | DSC med [Q1-Q3] | HD95 med [Q1-Q3] | DSC med [Q1-Q3] | HD95 med [Q1-Q3] | DSC med [Q1-Q3] | HD95 med [Q1-Q3] |
| *Segmentation with T2-FLAIR input* | | | | | | | | |
| r=0.0 | 91.0  [82.0-95.0] | 2.01  [1.07-3.46] | 94.0  [88.0-97.0] | 1.00  [1.00-1.41] | 91.0  [79.0-97.0] | 1.41  [1.00-4.12] | 92.0  [85.0-96.0] | 1.73  [1.00-3.46] |
| r=0.35 | 91.0  [81.0-95.0] | 2.00  [1.14-3.42] | 94.0  [87.0-97.0] | 1.00  [1.00-1.41] | 92.0  [79.0-97.0] | 1.41  [1.00-4.12] | 92.0  [85.0-96.0] | 1.41  [1.00-3.61] |
| r=0.5 | 90.0  [81.0-95.0] | 2.04  [1.14-3.58] | 94.0  [87.0-97.0] | 1.00  [1.00-1.41] | 91.0  [79.0-97.0] | 1.41  [1.00-4.24] | 91.0  [85.0-95.0] | 1.72  [1.00-3.61] |
| *Segmentation without T2-FLAIR input* | | | | | | | | |
| r=0.0 | 64.0  [55.0-71.0] | 9.17  [6.53-13.06] | 91.0  [84.0-95.0] | 1.41  [1.00-2.24] | 89.0  [72.0-96.0] | 2.24  [1.00-5.20] | 14.0  [3.0-28.0] | 22.44  [16.43-31.03] |
| r=0.35 | 89.0  [79.0-93.0] | 2.26  [1.41-4.06] | 94.0  [87.0-96.0] | 1.00  [1.00-1.41] | 92.0  [79.0-97.0] | 1.73  [1.00-4.24] | 87.0  [78.0-92.0] | 2.45  [1.73-4.69] |
| r=0.5 | 89.0  [79.0-94.0] | 2.30  [1.38-4.17] | 93.0  [87.0-96.0] | 1.00  [1.00-1.41] | 92.0  [76.0-97.0] | 1.73  [1.00-4.36] | 88.0  [79.0-92.0] | 2.24  [1.73-4.64] |

**Supplementary Table S1. UPenn-GBM external validation (n=403): Label-wise segmentation performance with T2-FLAIR present and absent at inference.** Performance of label-wise nnU-Net models (NCR/NET, ED, ET) trained on BraTS 2021 with targeted T2-FLAIR dropout probability rates of 0.0, 0.35, and 0.50 and evaluated under two prespecified inference scenarios: T2-FLAIR present and T2-FLAIR absent (T2-FLAIR channel replaced with zeros). Values are median [Q1-Q3] across patients. Overall denotes the unweighted macro-average across NCR/NET, ED, and ET.

**Abbreviations:** DSC = Dice similarity coefficient (reported as %); HD95 = 95th-percentile Hausdorff distance (mm); NCR/NET = necrotic and non-enhancing tumor; ED = edema; ET = enhancing tumor; FLAIR = fluid-attenuated inversion recovery; r = dropout rate.

|  | Median DSC r(0.35)-r(0) [95%CI] | TOST p-Value |
| --- | --- | --- |
| *Segmentation with T2-FLAIR input* | | |
| Overall | 0.0442 [0.002, 0.092] | <0.001 |
| ET | -0.0071 [-0.056, 0.027] | <0.001 |
| NET | 0.0320 [0.011, 0.074] | <0.001 |
| Edema | -0.0417 [-0.078, 0.029] | <0.001 |
| *Segmentation without T2-FLAIR input* | | |
| Overall | 24.1974 [23.613, 24.685] | 1 |
| ET | 1.075 [0.916, 1.241] | 1 |
| NET | 0.9508 [0.705, 1.213] | 1 |
| Edema | 69.3675 [67.202, 70.926] | 1 |

**Supplementary Table S2. UPenn-GBM external validation (n=403): Paired equivalence testing (TOST) for r=0.35 versus r=0.0 in label-wise models.** Label-wise models trained with targeted T2-FLAIR dropout (r=0.35) were compared with no-dropout models (r=0.0) under T2-FLAIR-present and T2-FLAIR-absent inference scenarios. Entries report median paired DSC differences (r=0.35 − r=0.0) in percentage points with 95% bootstrap confidence intervals. TOST p values test equivalence within prespecified bounds of ±1.5 DSC percentage points (α=0.05).

**Abbreviations:** TOST = two one-sided tests; DSC = Dice similarity coefficient; NCR/NET = necrotic and non-enhancing tumor; ED = edema; ET = enhancing tumor; FLAIR = fluid-attenuated inversion recovery; r = dropout rate.

|  | Median DSC r(0.35)-r(0.50) [95%CI] | TOST p-Value |
| --- | --- | --- |
| *Segmentation with T2-FLAIR input* | | |
| Overall | 0.075 [0.049, 0.093] | <0.001 |
| ET | 0.034 [0.003, 0.066] | <0.001 |
| TC | 0.049 [0.028, 0.067] | <0.001 |
| WT | 0.075 [0.038, 0.108] | <0.001 |
| *Segmentation without T2-FLAIR input* | | |
| Overall | -0.113 [-0.146, -0.076] | <0.001 |
| ET | -0.061 [-0.10, -0.006] | <0.001 |
| TC | -0.035 [-0.054, -0.018] | <0.001 |
| WT | -0.201 [-0.233, -0.140] | <0.001 |

**Supplementary Table S3. UPenn-GBM external validation (n=403): Paired equivalence testing (TOST) for r=0.35 versus r=0.50 in region-wise models.** Region-wise models trained with targeted T2-FLAIR dropout (r=0.35 and r=0.50) were compared under T2-FLAIR-present and T2-FLAIR-absent inference scenarios. Entries report median paired DSC differences (r=0.35 − r=0.50) in percentage points with 95% bootstrap confidence intervals. TOST p values test equivalence within prespecified bounds of ±1.5 DSC percentage points (α=0.05).

**Abbreviations:** TOST = two one-sided tests; DSC = Dice similarity coefficient; WT = whole tumor; TC = tumor core; ET = enhancing tumor; FLAIR = fluid-attenuated inversion recovery; r = dropout rate.

|  | Median DSC r(0.35)-r(0.50) [95%CI] | TOST p-Value |
| --- | --- | --- |
| *Segmentation with T2-FLAIR input* | | |
| Overall | 0.247 [0.183, 0.316] | <0.001 |
| ET | 0.188 [0.118, 0.233] | <0.001 |
| NET | 0.256 [0.171, 0.329] | <0.001 |
| Edema | 0.172 [0.122, 0.218] | <0.001 |
| *Segmentation without T2-FLAIR input* | | |
| Overall | -0.051 [-0.138, 0.013] | <0.001 |
| ET | -0.007 [-0.067, 0.041] | <0.001 |
| NET | 0.0484 [0.000, 0.136] | <0.001 |
| Edema | -0.234 [-0.301, -0.172] | <0.001 |

**Supplementary Table S4. UPenn-GBM external validation (n=403): Paired equivalence testing (TOST) for r=0.35 versus r=0.50 in label-wise models.** Label-wise models trained with targeted T2-FLAIR dropout (r=0.35 and r=0.50) were compared under T2-FLAIR-present and T2-FLAIR-absent inference scenarios. Entries report median paired DSC differences (r=0.35 − r=0.50) in percentage points with 95% bootstrap confidence intervals. TOST p values test equivalence within prespecified bounds of ±1.5 DSC percentage points (α=0.05).

**Abbreviations:** TOST = two one-sided tests; DSC = Dice similarity coefficient; NCR/NET = necrotic and non-enhancing tumor; ED = edema; ET = enhancing tumor; FLAIR = fluid-attenuated inversion recovery; r = dropout rate.

| BraTS Label/Region | HD-Glio Label | r=0.35 Median DSC (%) [Q1-Q3] | HD-GLIO Median DSC (%) [Q1-Q3] | Median Δ DSC (%) (model - HD) | Non-inferior p-Value |
| --- | --- | --- | --- | --- | --- |
| Edema | NE | 91.77 [84.91-95.55] | 87.68 [78.30-92.30] | 3.158 [2.733, 3.792] | <0.001 |
| ET | CE | 93.96 [87.49-96.80] | 90.31 [83.79-93.40] | 3.026 [2.698, 3.262] | <0.001 |
| WT | NE+CE | 95.65 [91.94-97.81] | 91.01 [84.77-94.24] | 4.087 [3.702, 4.360] | <0.001 |

**Supplementary Table S5. UPenn-GBM external validation (n=403): Noninferiority of the r=0.35 dropout model versus HD-GLIO in the T2-FLAIR-present scenario.** The targeted T2-FLAIR dropout model (r=0.35) was compared with HD-GLIO under the T2-FLAIR-present inference scenario. Endpoints were compared using the following prespecified correspondences: ET (BraTS) versus CE (HD-GLIO), ED (BraTS label-wise edema) versus NE (HD-GLIO), and WT (BraTS region-wise) versus NE+CE (HD-GLIO). Values are median DSC (%) [Q1-Q3]. Median ΔDSC denotes the paired median difference (model − HD-GLIO) in DSC percentage points. Noninferiority p values correspond to a one-sided test with a prespecified noninferiority margin of −1.5 DSC percentage points (α=0.05).

**Abbreviations:** DSC = Dice similarity coefficient; ET = enhancing tumor; ED = edema; WT = whole tumor; CE = contrast enhancing; NE = non-enhancing; r = dropout rate.

Supplementary material is not typeset so please ensure that all information is clearly presented, the appropriate caption is included in the file and not in the manuscript, and that the style conforms to the rest of the article.

# 1 Supplementary Figures and Tables

For more information on Supplementary Material and for details on the different file types accepted, please see [here](https://www.frontiersin.org/guidelines/author-guidelines#supplementary-material).

## 1.1 Supplementary Figures

**Supplementary Figure 1.** The figure legends are required to have the same font as the main text, 12 point normal Times New Roman, single spaced. Please use a single paragraph for each legend and prepare the figures keeping in mind the PDF layout.
